# HIV and intestinal parasite co-infections among a Chinese population: an immunological profile

**DOI:** 10.1186/2049-9957-2-18

**Published:** 2013-08-23

**Authors:** Li-Guang Tian, Tian-Ping Wang, Shan Lv, Feng-Feng Wang, Jian Guo, Xiao-Mei Yin, Yu-Chun Cai, Mary Kathryn Dickey, Peter Steinmann, Jia-Xu Chen

**Affiliations:** 1 National Institute of Parasitic Diseases, Chinese Center for Disease Control and Prevention (China CDC); WHO Collaborating Centre for Malaria, Schistosomiasis and Filariasis; Key Laboratory of Parasite and Vector Biology, Ministry of Health, Shanghai 200025, People’s Republic of China; 2 Anhui Institute of Parasitic Disease Control, Hefei 241000, People’s Republic of China; 3 University of Oklahoma, Midwest 73110, OK USA; 4 Department of Epidemiology and Public Health, Swiss Tropical and Public Health Institute, Basel 4051, Switzerland; 5 University of Basel, Basel 4051, Switzerland

**Keywords:** HIV, Intestinal parasite, Co-infection, Immunological profile, People’s Republic of China

## Abstract

**Background:**

Parasite infections often result in a switch of the human body’s predominant immune reaction from T-helper 1 (Th1)-type to Th2-type. Hence, parasite infections are widely expected to accelerate the progression of human immunodeficiency virus (HIV) infections to acquired immunodeficiency syndrome (AIDS). In the People’s Republic of China, both parasitic diseases and AIDS are epidemic in certain rural areas, and co-infections are relatively common. However, no population-based studies have yet investigated the frequency of HIV and parasite co-infections, and its effects on immune responses. We studied (1) the immune status of an HIV-infected population, and (2) the effect of co-infection of HIV and intestinal parasites on selected parameters of the human immune system.

**Methods:**

A total of 309 HIV-infected individuals were recruited and compared to an age-matched and sex-matched control group of 315 local HIV-negative individuals. Questionnaires were administered to all participants to obtain information on sociodemographic characteristics, sanitation habits, family income, and recent clinical manifestations. Two consecutive stool samples and 10 ml samples of venous blood were also collected from each individual for the diagnosis of parasite infections and quantitative measurements of selected cytokines and CD4+ T-lymphocytes, respectively.

**Results:**

During the study period, 79 HIV-infected individuals were not under highly active antiretroviral therapy (HAART) and were thus included in our analysis; the prevalence of intestinal helminth infections was 6.3% and that of protozoa was 22.8%. The most common protozoan infections were *Blastocystis hominis (B. hominis)* (13.9%) and *Cryptosporidium* spp. (10.1%). The prevalence of *Cryptosporidium* spp. in HIV-infected individuals was significantly higher than that in HIV negative individuals (P < 0.05). Compared to the non-co-infected population, no significant difference was found for any of the measured immunological indicators (P > 0.05). However, the following trends were observed: IFN-γ levels were lower, but the IL-4 level was higher, in the population co-infected with HIV and helminths. In the population co-infected with HIV and *B. hominis*, the IL-2 level was higher. The population co-infected with HIV and *Cryptosporidium* spp. had markedly lower CD4+ T-lymphocyte counts.

**Conclusion:**

According to the immunologic profile, co-infection with helminths is disadvantageous to HIV-infected individuals. It was associated with a shift in the Th1/Th2 balance in the same direction as that caused by the virus itself, which might indicate an acceleration of the progress from an HIV infection to AIDS. Co-infection with *Cryptosporidium* spp. was not associated with a significant change in immune factors but co-infection with *Cryptosporidium* spp. was associated with a reduced level of CD4 + T-lymphocytes, confirming the opportunistic nature of such infections. Co-infection with *B. hominis*, on the other hand, was associated with an antagonistic shift in the immunological profile compared to an HIV infection.

## Multilingual abstracts

Please see Additional file 1 for translations of the abstract into the six official working languages of the United Nations.

## Background

HIV/AIDS is a global health problem [1], as are parasite infections [2]. Co-infections of HIV and parasites including intestinal protozoa and helminths are particular concerns as both are rampant in low-income areas where the health status of the population has traditionally been low, even before the advent of HIV [3]. The detrimental health effects of intestinal parasite infections, such as chronic diarrhea, weight loss, and malnutrition might be major causes of death among AIDS patients [4]. HIV undermines the human immune system resulting in an increased chance of infection with certain opportunistic and other parasite species (e.g. *Cryptosporidium* spp., microsporidia, *Giardia intestinalis*, and *Strongyloides stercoralis*) [5-12]. HIV is also suspected to negatively impact the natural evolution of certain parasite infections and lead to more serious clinical symptoms, as well as complicating treatment [13,14]. On the other hand, the mucosal damage caused by many parasite infections could result in an increased susceptibility to HIV infections [15]. In addition, immunological changes caused by parasite infections may facilitate HIV replication and accelerate disease progression [16-18].

Immunological studies have shown that T-helper 1 (Th1)-type immune responses are associated with viral infections [16] while the immune response of the human body in the face of helminth infections often shifts from Th1-type to Th2-type [19]. Since Th1 cells are necessary to control HIV, parasite infections are suspected to accelerate the progression of HIV infections to AIDS [20,21]. It is thus of great importance to understand the immune response of individuals co-infected with HIV and parasitic infections in order to predict the course of infection in co-infected individuals. This would allow HIV infections to be managed more effectively, contributing to an improved quality of life for HIV patients.

In the People’s Republic of China (P.R. China), both intestinal parasitic diseases and AIDS are endemic in rural areas [22-26]. Therefore, co-infections of HIV and intestinal parasites are likely, however, the phenomenon has not really been studied in detail. Results from our investigations show that the prevalence of intestinal protozoa is as high as 23.2% among HIV-infected individuals in a typical rural setting [27]. However, no population-based studies on HIV and parasite co-infections and its effects on immune responses have yet been conducted in the country [27]. The objectives of this study were to: (1) reveal the immune status of an HIV-infected population; and (2) evaluate the effects of HIV and intestinal parasite co-infections on selected parameters of the human immune system.

## Methods

### Study population

The study was conducted in Fuyang in the Anhui Province in P.R. China between June and August 2008. The criteria for participants were: (1) 6–65 years of age, (2) ability to give written informed consent or to obtain assent by legal guardians, and (3) absence of obvious mental illnesses or other diseases affecting investigation procedures.

HIV-infected individuals registered with local and provincial health departments were contacted for inclusion in the study. A control group of HIV-negative individuals residing in the same communities was then identified, matched by age and sex. The control group was recruited from family members of HIV-infected study participants and, if no suitable controls were available, from people in their neighborhood.

### Questionnaire and sample collection

Questionnaires were administered to all study participants by health professionals from the local Center for Disease Control and Prevention (CDC). All interviewers had training for routine tasks related to HIV care and prevention. Questions focused on sociodemographic characteristics, sanitation habits, family income, and recent clinical manifestations. One fresh stool samples was collected from each study participant over several days, with a second sample collected whenever possible to maximize the sensitivity of the diagnostic approaches (maximum one sample/day). A 10-ml venous blood sample was also obtained from each study participant.

### Laboratory tests

Current HIV – and intestinal parasite infection status, cytokine concentration, and CD4+ T-lymphocyte counts – were determined for all study participants. HIV infections were diagnosed using an enzyme-linked immunosorbent assay (ELISA; Beijing Jinhao Biologic Medicine Company, P.R. China). The critical cutoff value was calculated as the mean optical density (OD) value of the positive controls multiply by 10%. Individuals with an OD value below the critical value were considered HIV negative. ELISA-positive samples were subject to confirmation by Western blot (HIV Blot 2.2 WB; Genelabs Diagnostics, Singapore). Individuals with two positive test results were confirmed as HIV positive. Both tests were conducted in the local CDC station.

The CD4+ T-lymphocyte counts were determined using FACSCalibur flow cytometry (Becton Dickinson, USA). Quantitative ELISA kits (Shanghai Fuzhong Science and Technology Company, P.R. China) were used to measure cytokine concentrations in the serum. Tested cytokines included IL-2, IL-4, IL-10, and IFN-γ. Both CD4+ T-lymphocyte and cytokine measurements were completed by staff of the National Institute of Parasitic Diseases in Shanghai and the Institute of Parasitic Diseases of the Anhui Province.

The Kato-Katz [28] and charcoal culture methods [29] were used to diagnose intestinal helminth infections. The focus was on detecting common soil-transmitted helminths (*Ascaris lumbricoides*, hookworm, *Trichuris trichiura*), *Clonorchis sinensis*, and *Strongyloides stercoralis*. Eggs of the former four species were identified morphologically by examining three Kato-Katz slides from each stool sample while *S. stercoralis* larvae were detected by the charcoal culture method. For the diagnosis of protozoa, an in vitro culture method [30] was employed to detect *B. hominis* while *Cryptosporidium* spp. was detected by modified acid-fast staining [31]. *G. intestinalis* and *Entamoeba* spp. were detected by the Lugol’s iodine method [22]. A positive result in any of the two samples submitted by an individual was sufficient to diagnose an infection.

### Statistical analysis

All data were entered twice into a database established using EpiData 3.1. The Student’s *t*-test, Dunnett *t* test, variance analysis, and *χ*^2^ testing were used for statistical data analysis in SAS9.1, as appropriate.

An optimized immune function model (1) [32] was used to analyze changes in immune response and synergistic effects.

(1) $$T=\frac{\mathrm{IFN}‒\gamma }{\left(\mathrm{IFN}‒\gamma +\mathrm{IL}‒4\right)}$$

Here, *T* is defined as the proportion of cytokines associated with Th1-type cells in the total cytokine pool. *IFN-γ* is considered as an indicator of Th1 responses and *IL-4* as an indicator of Th2 responses.

### Ethical considerations

The study protocol was approved by the institutional review board of the National Institute of Parasitic Diseases, Chinese Center for Disease Control and Prevention in Shanghai. Potential study participants were contacted through the village leaders, and the objectives, procedures, and potential risks were carefully explained. Interested individuals provided written informed consent in person or, in the case of minors, assent was granted by parents or legal guardians before enrolment into the study. All participants were offered professional counseling before and after HIV testing by staff of the local AIDS prevention and treatment agencies where they were enrolled for free treatment and follow-up. Free anthelmintics (albendazole, praziquantel) were offered to all participants found to be infected with helminths through local healthcare institutions. All diagnostic results and other data collected in the frame of the study were strictly confidential.

## Results

### Participation and demographic information

**Figure 1:**
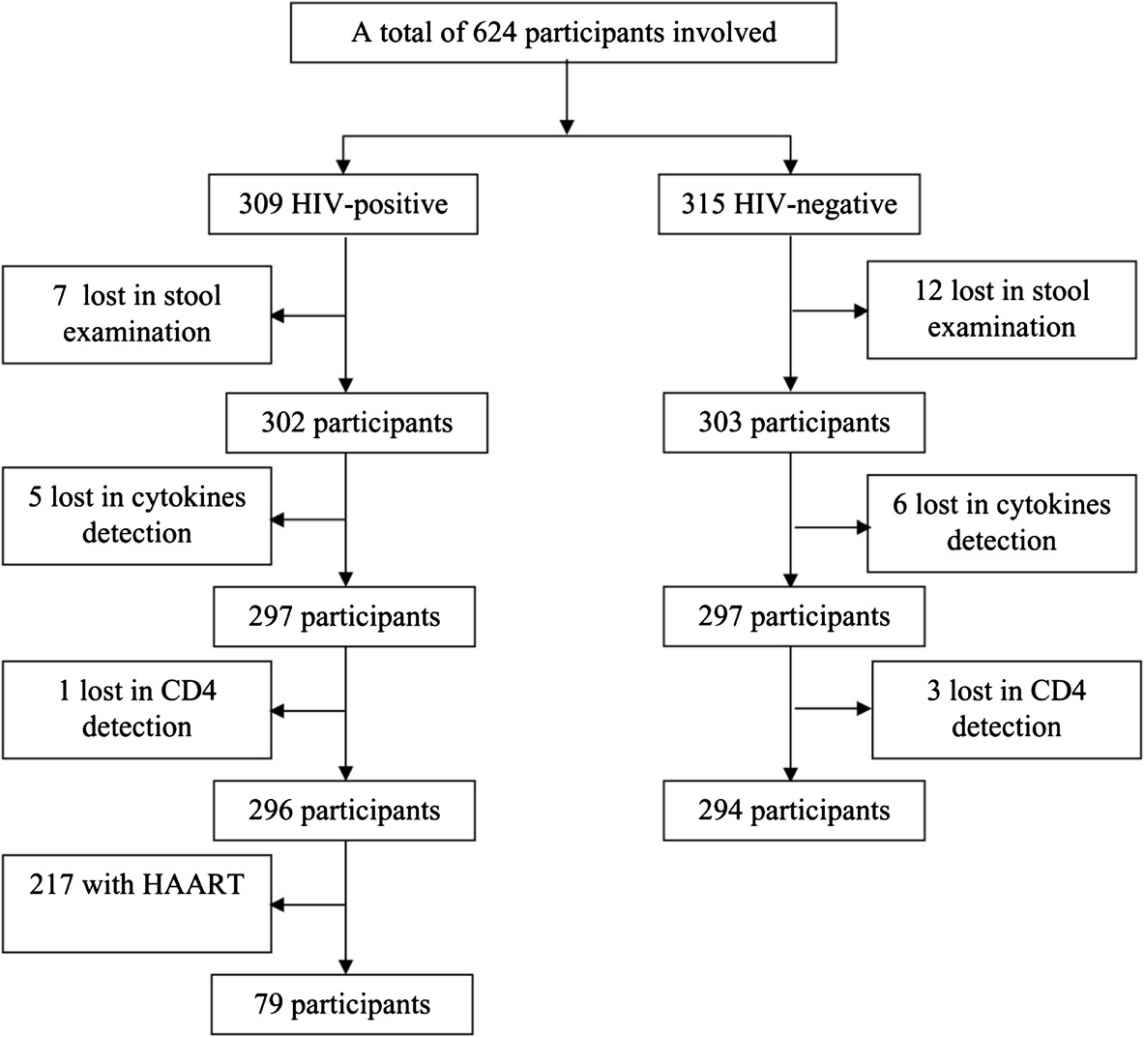
Number of study participants at various stages of recruitment.

A total of 624 individuals were enrolled into the study. Among them, 590 individuals (278 males and 312 females; 296 HIV-infected and 294 HIV-negative) provided samples of sufficient quality and quantity to perform all three examinations, i.e. parasite diagnosis in stool, and cytokine and CD4+ T-lymphocyte quantification in blood (see Figure 1). The 217 HIV-infected individuals that were at that time undergoing highly active antiretroviral therapy (HAART) were excluded from analyses focusing on immune functions as the treatment affected their immune response. Therefore, only 79 out of 296 HIV-infected individuals were included in relevant subsequent analyses. The average age of the study participants was 42.5 years – 42.8 years for the HIV-infected group (n = 79) and 41.6 years for the HIV-negative group (n = 294).

### Parasite infections

Among the 79 HIV-infected individuals who were not on HAART, the overall prevalence of helminth infections was 6.3% (5/79) and that of protozoa infection was 22.8% (18/79). *B. hominis* and *Cryptosporidium* spp. were the most common species: their prevalence was 13.9% (11/79) and 10.1% (8/79), respectively. Three individuals were infected with both *B. hominis* and *Cryptosporidium* spp.

Among the 294 HIV-negative individuals, 5.4% (16/294) were infected with helminthes and 24.8% (73/294) with protozoa. Both prevalences were not significantly different from the recorded value among the HIV-infected population. Five individuals were infected with both helminths and protozoa. The prevalence of *B. hominis* in the HIV-negative population was markedly higher (21.8%) than that in the HIV-infected population (13.9%), whereas that of *Cryptosporidium* spp. was significantly lower in the HIV-negative population (3.1%) compared to the HIV-infected population (10.1%) (P = 0.0134). The parasite infections in the HIV-positive and -negative populations are shown in Table 1.

**Table 1:** Prevalence of parasitic infections among HIV-infected and HIV-negative study participants

**Variate**	**HIV infected (n = 79)% (No. positive)**	**HIV negative (n = 294)% (No. positive)**	**P value**
*Ascaris lumbricoides*	0	0.68 (2)	1.0000*
Hookworm	6.33 (5)	4.42 (13)	0.5548*
*Trichuris trichiura*	0	0.34 (1)	1.0000*
*Clonorchis sinensis*	0	0.68 (2)	1.0000*
Any helminthes	6.33 (5)	5.44 (16)	0.7840*
*Giardia intestinalis*	1.26 (1)	0.34 (1)	0.3828*
*Blastocystis hominis*	13.92 (11)	21.77 (64)	0.1225
*Entamoebae spp.*	3.80 (3)	0.68 (2)	0.2914*
*Cryptosporidium spp.*	10.13( 8)	3.06 (9)	0.0134*
Any protozoa	22.78 (18)	24.83 (73)	0.7071

*tested by the Fisher exact test, others tested by the chi-square test.

### Immune factor profile

The major differences between immune factors in the HIV-positive and -negative populations are shown in Table 2. Compared to the HIV-negative group (294 individuals), the average count of CD4+ T-lymphocytes in the HIV-positive group (79 individuals) was significantly lower (P < 0.0001). The level of IL-2 and IL-4 was higher in the HIV-infected group compared to the HIV-negative control group. However, IFN-γ and IL-10 levels were not significantly different in the two groups. As a result, the *T* value of the HIV-positive group (0.35) is significantly lower than that of the in HIV-negative group (0.45). The pattern remained the same if participants with parasitic infections were excluded from the analysis (second column in Tables 3 and 4).

**Table 2:** Levels of selected immunological indicators in HIV-infected and HIV-negative study participants

	**HIV-infected (n = 79)** $\left(\bar{x}\pm s\right)$	**HIV negative (n = 294)** $\left(\bar{x}\pm s\right)$	**P value**
CD4+	518.12 ± 253.56	829.89 ± 261.77	<.0001
IL-2	87.92 ±57.13	57.19 ± 41.29	<.0001*
IL-4	25.06 ±17.15	14.72 ± 8.52	<.0001*
IL-10	20.79 ±25.70	25.59 ± 47.72	0.2330*
IFN-γ	12.58 ±7.69	12.37 ± 7.65	0.8236
T**	0.35 ±0.17	0.45 ± 0.19	<.0001

*variance was uneven; using the Satterthwaite test.

**T = IFN-γ/(IFN-γ + IL-4).

**Table 3:** Levels of selected immunological indicators in HIV-negative individuals, stratified by parasitic infection status

	**Without parasite infection**	**Only helminth infections**	**Only B. hominis infection**	**Only Cryptosporidium spp. infection**	**Both helminth and protozoan infections**	P-Value**
	**(n = 210)** $\left(\bar{x}\pm s\right)$	**(n = 11)** $\left(\bar{x}\pm s\right)$	**(n = 56)** $\left(\bar{x}\pm s\right)$	**(n = 9)** $\left(\bar{x}\pm s\right)$	**(n = 5)** $\left(\bar{x}\pm s\right)$	
CD4+	842.2 ± 270.5	802.5 ± 219.8	794.6 ± 249.6	739.8 ± 175.9	937.6 ± 251.9	0.4796
IL-2	56.4 ± 41.4	69.8 ± 49.0	55.6 ± 40.3	64.2 ± 45.2	58.9 ± 46.7	0.8432
IL-4	14.8 ± 9.0	15.5 ± 6.3	14.3 ± 6.6	15.1 ± 12.0	13.0 ± 6.6	0.9801
IL-10	25.4 ± 51.0	22.1 ± 25.1	28.5 ± 43.0	27.6 ± 38.6	8.3 ± 5.3	0.9212
IFN-γ	12.3 ± 7.2	13.8 ± 12.5	12.0 ± 7.0	11.2 ± 5.5	10.1 ± 2.9	0.8762
T*	0.5 ± 0.2	0.4 ± 0.2	0.4 ± 0.2	0.5 ± 0.3	0.5 ± 0.2	0.9135

* *T* = IFN-γ/(IFN-γ + IL-4).

**Dunnett *t* test was applied in paired comparison among groups and group 1 was the control group; P > 0.05.

**Table 4:** Levels of selected immunological indicators in HIV-infected individuals, stratified by parasitic infection status

	**Without parasitic infection**	**Only helminth infections**	**Only B. hominis infection**	**Only Cryptosporidium. spp. infection**	P-Value**
	**(n = 56)** $\left(\bar{x}\pm s\right)$	**(n = 5)** $\left(\bar{x}\pm s\right)$	**(n = 8)** $\left(\bar{x}\pm s\right)$	**(n = 7)** $\left(\bar{x}\pm s\right)$	
CD4+	520.3 ± 238.7	420.8 ± 262.1	668.0 ± 401.8	411.0 ± 141.5	0.2006
IL-2	82.6 ± 53.8	70.6 ± 65.8	121.3 ± 68.4	100.8 ± 69.4	0.2725
IL-4	23.6 ± 17.6	23.8 ± 22.1	28.6 ± 15.5	27.1 ± 14.0	0.8599
IL-10	22.2 ± 29.8	18.3 ± 7.0	19.0 ± 9.4	18.5 ± 12.8	0.9655
IFN-γ	12.5 ± 6.7	7.5 ± 5.7	12.4 ± 4.7	16.2 ± 11.4	0.3078
T*	0.4 ± 0.2	0.3 ± 0.2	0.3 ± 0.2	0.4 ± 0.2	0.7041

* *T* = IFN-γ/(IFN-γ + IL-4).

**Dunnett *t* test was applied in paired comparison among groups and group 1 was the control group; P > 0.05.

### Immune factors and parasite infection status

In both HIV-infected individuals and those who were HIV negative, no significant difference was found for any of the measured immunological indicators in people with different parasite infections (see Tables 3 and 4). However, a trend towards lower IFN-γlevels in individuals co-infected with HIV and helminths was observed, albeit with marked variation between individual values. In contrast, the level was elevated in individuals co-infected with HIV and *Cryptosporidium* spp. The IL-4 level in HIV-infected individuals was generally higher than that in HIV-negative controls regardless of parasite infection status. While the IL-4 level was stable across the HIV-negative population, it appeared to fluctuate in the HIV-infected population according to their infection status with different parasite species. For example, the level was higher when co-infection of HIV and *B. hominis* was present. As a result, the *T* value was lower in HIV-positive individuals than in the HIV-negative control group, indicating that the immune responses in HIV-infected individuals co-infected with worms was predominantly of Th2 type.

The IL-2 level in the HIV-infected population was generally higher than in the HIV-negative control group. However, no significant difference between these two groups was observed in the helminth-infected subpopulation. Of note, in the HIV-infected group, the IL-2 level was higher in the case of co-infection with *Cryptosporidium* spp. or *B. hominis*. However, in the HIV-negative control group, the IL-2 level was not markedly different if an infection with *Cryptosporidium* spp. or *B. hominis* was present*.*

IL-10 levels were slightly lower in the HIV-infected study participants compared to the HIV-negative control group. The difference considerably increased when *B. hominis* and *Cryptosporidium* spp. were involved.

CD4+ T-lymphocyte levels were significantly lower in the HIV-positive individuals (P < 0.01) compared to their HIV-negative counterparts. Among the individuals co-infected with HIV and helminths or *Cryptosporidium* spp., CD4+ T-lymphocyte levels were even lower. In contrast, co-infection of HIV and *B. hominis* was associated with higher CD4+ T lymphocyte levels.

## Discussion and conclusions

It is now widely accepted that by undermining the human immune response, HIV infections facilitate the establishment of opportunistic parasite infections such as *Pneumocystis* spp., microsporidia, and *Cryptosporidium* spp. [33-35]. Indeed, our findings show that the prevalence of *Cryptosporidium* spp. in the HIV-infected population was 10.1%, significantly higher than that of 3.1% among HIV-negative group. The immune profile of HIV-infected individuals was markedly different from that of their HIV-negative peers. However, no significant change in immune system indicators was observed when individuals with HIV infections but no parasite co-infection were compared to those with such co-infections. *Cryptosporidium* spp. infections in HIV-infected individuals often cause severe diarrhea and malnutrition, and can even lead to death among AIDS patients [36].

*B. hominis* is one of the most common human intestinal parasite infections. *B. hominis* is usually considered a commensal (non-pathogenic) protozoa inhabiting the human intestine, although previous studies have suggested that it has certain pathogenicity [37-39]. Interestingly, the prevalence of *B. hominis* among HIV-infected individuals was actually lower than that among the controls in our study.

The appropriate immune reaction against helminth infections is believed to be provided by Th2 cells which inhibit the development of Th1 cells, and hence prevent them and macrophages from mounting massive immune reactions against the comparatively very large parasites – a reaction which would potentially be harmful for the human body [40,41]. The progression from HIV infection to AIDS is characterized by a decrease of Th1-type immune responses whereas Th2-type responses increase [42-44]. In our study, 6.3% of the HIV-infected participants were co-infected with intestinal helminthes, and the level of IFN-γ and IL-10 was lower among these co-infected individuals while the level of IL-4 was higher. The Th2-type immune response was elevated and the T value (T = Th1 / (Th1 + Th2) decreased. Thus, the co-infection of HIV and helminths acted synergistically in shifting the Th1/Th2 balance which might indicate an acceleration of the progress from HIV infection to AIDS. It was further observed that in individuals with HIV and *B. hominis* co-infection, CD4 levels were slightly lower compared to individuals without a *B. hominis* infection, while IFN-γ and other cytokine levels did not change significantly and IL-2 levels were higher. IL-2 is indispensable for cell-mediated immune responses against parasitic infections and humoral immunity. Therefore, the presence of *B. hominis* may even be favorable for HIV-positive individuals as IL-2 induces T cells to secrete IFN-γ, and the latter is an important factor in anti-viral immune reactions. The promotion of human B cell proliferation should inhibit IL-4. However, in our study IL-4 levels were found to be slightly higher. To sufficiently determine the reasons, further studies need to be conducted. The IL-10 level was slightly lower if co-infection of HIV and *B. hominis* was present. IL-10 can inhibit Th1-stimulating cytokine secretion. Together, these findings indicate, once again, a need to further study the pathogenicity of *B. hominis* in order to better determine its status as a member of the intestinal flora [45-47].

In conclusion, co-infection with helminths was associated with potentially detrimental changes of the immunologic profile of HIV-infected individuals. Helminth infections appeared to act synergistically with HIV in shifting the Th1/Th2 balance, a shift which has traditionally be seen as indicative of an acceleration of the progress from HIV infection to AIDS. On the other hand, co-infection with protozoa was imperceptible in the immune response profile: co-infection with *Cryptosporidium* spp. was not associated with any significant change in studied immune factors while co-infection with *B. hominis* was associated with favorable shifts in the immune profile.

## Competing interests

The authors declare that they have no competing interests.

## Authors’ contributions

LGT conceived the study, collected the data and analyzed it, and drafted the manuscript. SL and TPW conceived the project, assisted in the interpretation of the results, and revised the manuscript. MKD and PS revised the manuscript and provided intellectual input for the interpretation of the findings. FFW, JG, XMY, and YCC conceived the project and provided technical support for data collection and analysis. JXC conceived the study and revised the manuscript. All authors read and approved the final manuscript.

## Supplementary Material

Additional file 1 Multilingual abstracts in the six official working languages of the United Nations. Click here for file
