# Supplementary material for: HIV and intestinal parasite co-infections among a Chinese population: an immunological profile
Source: Infect Dis Poverty. 2013 Aug 23;2:18. doi: 10.1186/2049-9957-2-18 (PMC3766051; doi:10.1186/2049-9957-2-18)
Supplement: Additional file 1 — Multilingual abstracts in the six official working languages of the United Nations. [file 2049-9957-2-18-S1.pdf]

Translation of the abstract into the six official working languages of the United Nations

فيروس العوز المناعي البشري وعدوى الطفيليات المعوية المرافقة في سكان صينيين: ملف مناعي

لي – جوانج تيان، تيان – بينج وانج، شوان لف، فينج – فينج وانج، جيان جو، شيوا- مي ين، يون – تشون كاي، ماري كاثرين ديكي، بيتر شتينمان و جيا – زو تشين

ملخص

خلفية: عادة ما تنتج العدوى الطفيلية من تحول رد فعل مناعة الجسم البشري السائدة من نوع (Th1) إلى النوع (Th2). ومن ثم، فإن عدوى الطفيليات تكون متوقعة بصورة كبيرة لتسريع تقدم عدوى فيروس العوز المناعي البشري إلى عرض نقص المناعة المكتسب (AIDS). وفي جمهورية الصين الشعبية، يُعتبر كلٌ من الأمراض الطفيلية وعرض نقص المناعة المكتسب (AIDS) أمراض وبائية في بعض المناطق الريفية وتكون العدوى المرافقة شائعة. ولكن، لا توجد دراسات قائمة على السكان قامت ببحث تكرار فيروس العوز المناعي والعدوى الطفيلية المرافقة، وتأثيراتها على الاستجابات المناعية. لقد قمنا بدراسة (1) الحالة المناعية للسكان المصابين بفيروس العوز المناعي و(2) تأثير العدوى المرافقة لفيروس العوز المناعي والطفيليات المعوية على الوسائط المختارة من نظام المناعة البشري.

الطريقة: تطوع 309 مريض بفيروس العوز المناعي وتمت مقارنتهم بمجموعة ضابطة تتكون من 315 من السكان المحليين الذين لا يعانون من فيروس العوز المناعي وتتطابق في السن والجنس. تم توزيع استبيانات على كافة المشاركين للحصول على معلومات عن الخصائص الاجتماعية والديمغرافية والعادات الصحية ودخل الأسرة والتجليات العيادية الأخيرة. كما تم جمع عينتين براز متتالية وعينات 10 مل دماء من الوريد من كل فرد لتشخيص العدوى الطفيلية والقياسات الكمية للسيتوكين والخلايا الليمفاوية -T +CD4 المختارة على التوالي.

النتائج: خلال فترة الدراسة، لم يكن 79 فرد مصاب بفيروس العوز المناعي يخضعون لمضادات فيروسات قهرية عالية النشاط (HAART) ولهذا تم ضمهم لتحليلنا؛ كان انتشار عدوى الإصابة الديدان الطفيلية المعوية هيلمينث 6.3% والإصابة بالحيوانات الأولية 22.8%. وكانت أكثر الإصابات بالحيوانات الأولية انتشاراً هي بلاستوسيستس هومينيس (B. hominis) بنسبة (13.9%) و كرايتوسبورديم سب بنسبة (10.1%). وكان انتشار الإصابة بكرايتوسبورديم سب. بين المصابين بفيروس العوز المناعي أعلى بصورة ملحوظة عن انتشاره بين الأفراد غير المصابين بفيروس العوز المناعي (P<.0.05). مقارنة بالسكان غير المصابين بالعدوى المرافقة، لا يوجد اختلاف ملحوظ لأي من المؤشرات المناعية المدروسة (P<.0.05). ولكن، تم ملاحظة الاتجاهات التالية: مستويات IFN-γ كانت أكثر انخفاضاً، ولكن مستوى IL-4 كان أعلى، بين الأفراد المصابين بالعدوى المرافقة بفيروس العوز المناعي ودودة هيلمينث. وفي الأفراد المصابين بالعدوى المرافقة بفيروس العوز المناعي ودودة ب. هومينيس، كان مستوى IL-2 أعلى. وقد سجل الأفراد المصابون بالعدوى المرافقة بفيروس العوز المناعي ودودة كرايتوسبورديم سب CD4 + T خلايا ليمفاوية أقل.

الاستنتاج: وفقاً لملف المناعة، فإن العدوى المرافقة بدودة هيلمينث غير ملائمة للأفراد المصابين بالعدوى المرافقة بفيروس العوز المناعي. وهي ترتبط بالتحول في مستوى Th1/ Th2 في الاتجاه ذاته كما لو كان المتسبب بها هو الفيروس نفسه،

وهو الأمر الذي يشير إلى تسريع تقدم عدوى فيروس العوز المناعي إلى عرض نقص المناعة المكتسب (AIDS). لم تكن العدوى المرافقة بكرايتوسبورديم سب مرتبطة بمستوى CD4+T – الغدد اللمفاوية المنخفض، تأكيداً للطبيعة الاستغلالية لتلك العدوى. ومن ناحية أخرى، فإن العدوى المرافقة بدودة هومينيس ب، كانت مرتبطة بالتحول المضاد في الملف المناعي مقارنة بعدوى فيروس العوز المناعي.

Translated from English version into Arabic by Reham Hussien, through

TRANSLATORS
WITHOUT BORDERS

合并肠道寄生虫感染对 HIV 感染者免疫功能的影响

田利光，汪天平，吕山，汪峰峰，郭俭，尹晓梅，蔡玉春，Mary Kathryn Dickey, Peter Steinmann，陈家旭

摘要

引言：寄生虫感染会破坏人体内 Th1/Th2 免疫平衡，加速 HIV 感染者转变为艾滋病患者的进程。在中国艾滋病流行严重的农村地区，HIV 阳性人群合并肠道寄生虫感染的情况非常普遍，本研究通过开展 HIV 和肠道寄生虫合并感染情况的调查，了解合并肠道寄生虫感染对 HIV 感染者免疫功能及病程的影响。

方法：现场招募 309 名 HIV 感染者和 315 名 HIV 阴性人群，通过问卷调查收集社会人口学、家庭收入、卫生设施、生活习惯以及临床症状等资料。同时采集粪便样本和血液样本，检测肠道寄生虫感染情况、体内各细胞因子水平以及 CD4+T 淋巴细胞数量。

结果：309 名 HIV 感染者中有 79 人未接受抗病毒治疗，其合并肠道蠕虫感染率为 6.3%，合并肠道原虫感染率为 22.8%，最常见的原虫感染为人芽囊原虫感染（13.9%）和隐孢子虫感染（10.1%），HIV 感染者隐孢子虫感染率显著高于 HIV 阴性人群隐孢子虫感染率（$P<0.05$）。合并肠道寄生虫感染者体内各细胞因子水平与未合并肠道寄生虫感染者相比无统计学差异，但以可以观察到以下结果：合并肠道蠕虫感染者体内 IFN-γ 水平较低，IL-4 水平较高，合并人芽囊原虫感染者体内 IL-2 水平较高，合并隐孢子虫感染者体内 CD4+ T 淋巴细胞数量较低。

结论：合并肠道蠕虫感染会造成体内 Th1/Th2 免疫平衡改变，加速 HIV 感染者进展为艾滋病患者的进程。合并隐孢子虫感染者体内 CD4+ T 淋巴细胞数量较低，而合并人芽囊原虫感染对 HIV 感染者病程进展无不利影响。

Translated from English version into Chinese by Li-Guang Tian

Co-infections par le VIH et les parasites intestinaux dans une population chinoise: profil immunologique

Li-Guang Tian, Tian-Ping Wang, Shan Lv, Feng-Feng Wang, Jian Guo, Xiao-Mei Yin, Yu-Chun Cai, Mary Kathryn Dickey, Peter Steinmann et Jia-Xu Chen

Résumé

Contexte : Les parasitoses provoquent souvent un changement de la réaction immunitaire prédominante du corps humain du type médié par les lymphocytes auxiliaires Th1 à celui médié par les Th2. Il est donc très probable qu'elles accélèrent la progression de l'infection par le virus de l'immunodéficience humain (VIH) vers le syndrome d'immunodéficience acquise (SIDA). Les parasitoses et le SIDA sont épidémiques dans certaines régions rurales de la République populaire de Chine, et les co-infections sont relativement fréquentes. Aucune étude de population n'avait pourtant été entreprise jusqu'ici pour évaluer la fréquence des co-infections par le VIH et les parasites et leurs effets sur la réponse immunitaire. Nous avons étudié (1) le statut immunitaire de la population infectée par le VIH et (2) l'effet sur certains paramètres immunitaires d'une co-infection par le VIH et des parasites internes.

Méthodes : Au total, 309 sujets infectés par le VIH ont été recrutés et comparés à un groupe témoin de 315 individus séronégatifs appartenant à la population locale, comparable en termes d'âge et de sexe. Des questionnaires ont été soumis à tous les participants afin de recueillir des informations sur les caractéristiques sociodémographiques, les habitudes sanitaires, le revenu familial et les manifestations cliniques récentes. Deux échantillons de selles consécutifs et des échantillons de sang veineux de 10 ml ont été recueillis obtenus de chaque individu afin de diagnostiquer les parasitoses et de quantifier certaines cytokines et les lymphocytes CD4+, respectivement.

Résultats : Au cours de la durée de l'étude, 79 sujets infectés par le VIH ne suivaient pas un traitement antirétroviral à haute activité (HAART) ; ceux-ci ont donc été inclus dans notre analyse. La prévalence des helminthiases intestinales était de 6,3 % et celle des protozooses de 22,8 %. Les protozoaires parasites les plus fréquents étaient *Blastocystis hominis (B. hominis)* (13,9 %) et *Cryptosporidium* spp. (10,1 %). La prévalence de *Cryptosporidium* spp. était significativement plus élevée chez les sujets infectés par le VIH que chez les sujets séronégatifs ($P<0,05$). Par rapport à la population non infectée, nous n'avons relevé aucune

différence dans les indicateurs immunologiques mesurés ($P > 0,05$). Les tendances suivantes ont néanmoins été observées : les taux d'IFN-γ étaient plus bas mais ceux d'IL-4 plus élevés dans la population co-infectée par le VIH et les helminthes. Dans celle co-infectée par le VIH et *B. hominis*, c'est le taux d'IL-2 qui était plus élevé. Enfin, la population co-infectée par le VIH et *Cryptosporidium* spp. affichait une numération des lymphocytes T CD4+ sensiblement plus basse.

Conclusion : Selon le profil immunologique, la coexistence d'une helminthiase est défavorable aux sujets infectés par le VIH. Nous avons constaté qu'elle était associée à un décalage de l'équilibre Th1/Th2 dans le même sens que celui causé par le virus lui-même, ce qui pourrait indiquer une accélération de la progression de l'infection par le VIH vers le SIDA. La co-infection par *Cryptosporidium* spp. n'est pas associée à une modification significative des facteurs immunitaires, à la différence de la co-infection par *Cryptosporidium* spp. qui est associée à une baisse de la numération de lymphocytes T CD4+, ce qui confirme le caractère opportuniste de ces parasitoses. En revanche, la co-infection par *B. hominis* s'est avérée associée à un décalage antagonique du profil immunologique par rapport à l'infection par le VIH.

Translated from English version into French by Suzanne Assenat, through

TRANSLATORS
WITHOUT BORDERS

Коинфицирование ВИЧ-инфекцией и кишечными паразитами среди населения Китая: иммунологический профиль

Ли-Гуаньг Тиан, Тиан-Пинг Ванг, Шан Лв, Фенг-Фенг Ванг, Джиан Гуо, Ксяо-Мей Йин, Йю-Чун Сай, Мери Кэтрин Дики, Питер Стайманн и Джиа-Ксу Чен

Аннотация

Краткое описание: Заражение паразитами часто приводит к тому, что преобладающая в организме человека иммунная реакция переключается с Т-хелперов 1-го типа (Th1) на тип Th2. Вследствие этого широко распространено мнение, что заражение паразитами ускоряет развитие инфекции вируса иммунодефицита человека (ВИЧ) в синдром приобретенного иммунодефицита (СПИД). В некоторых сельских районах Китайской Народной Республике заболевание паразитарными болезнями и СПИДом является эпидемическим и коинфицирование достаточно распространено. Однако, до сих пор не проводились популяционные исследования частоты коинфицирования ВИЧ-инфекцией и кишечными паразитами, а также влияния коинфицирования на иммунную реакцию. Мы изучали (1) иммунное состояние ВИЧ-инфицированного населения, и (2) влияние коинфицирования ВИЧ-инфекцией и кишечными паразитами на выбранные параметры иммунной системы человека.

Методы: В исследование были вовлечены 309 ВИЧ-инфицированных лиц. Было проведено их сравнение по возрасту и полу с контрольной группой из 315 ВИЧ-отрицательных местных жителей. Всем участникам были выданы опросные анкеты для сбора социально-демографических данных, сведений о санитарных условиях, семейном доходе и недавних клинических проявлениях заболеваний. У каждого участника были взяты два последовательных образца стула и по 10 мл венозной крови для анализа на паразитные инфекции и количественной оценки выбранных цитокинов и CD4+ Т-лимфоцитов соответственно.

Результаты: Во время проведения исследования 79 ВИЧ-инфицированных лиц не подвергались высокоактивной антиретровирусной терапии (ВААРТ) и были таким образом включены в наш анализ; распространенность кишечных глистных инфекций составила 6,3% и протозойных инфекций 22,8%. Наиболее часто встречающимися протозойными инфекциями были *Blastocystis hominis (B. hominis)* (13,9%) и

Cryptosporidium spp. (10,1%). Распространенность *Cryptosporidium* spp. среди ВИЧ-инфицированных лиц была значительно выше, чем среди ВИЧ-отрицательных лиц ($P<0,05$). По сравнению с некоинфицированным населением не было обнаружено существенных различий ни по одному из измеренных иммунологических показателей ($P>0,05$). Однако, были замечены следующие закономерности: у населения, коинфицированного ВИЧ-инфекцией и глистами, уровни IFN-γ были ниже, а уровень IL-4 выше. У населения, коинфицированного ВИЧ-инфекцией и *B. hominis*, уровень IL-2 был выше. У населения, коинфицированного ВИЧ-инфекцией и *Cryptosporidium* spp., содержание CD4+ Т-лимфоцитов было значительно ниже.

Заключение: Согласно иммунологическому профилю, коинфицирование глистами является неблагоприятным для ВИЧ-инфицированных лиц. Это связано со смещением баланса Th1/Th2 в том же направлении, в котором действует сам вирус, что может указывать на ускорение перехода ВИЧ-инфекции в СПИД. Коинфицирование *Cryptosporidium* spp. не связано со значительным изменением иммунных факторов, зато оно связано с пониженным уровнем CD4+ Т-лимфоцитов, что подтверждает оппортунистический характер таких инфекций. С другой стороны, коинфицирование *B. hominis* связано с антагонистическим сдвигом в иммунологическом профиле по сравнению с ВИЧ-инфекцией.

Translated from English version into Russian by Natalia Potashnik, through

TRANSLATORS
WITHOUT BORDERS

VIH y co-infecciones de parásitos intestinales en una población china: un perfil inmunológico

Li-Guang Tian, Tian-Ping Wang, Shan Lv, Feng-Feng Wang, Jian Guo, Xiao-Mei Yin, Yu-Chun Cai, Mary Kathryn Dickey, Peter Steinmann and Jia-Xu Chen

Abstracto

Antecedentes: Las infecciones parasitarias con frecuencia llevan a un cambio en la reacción inmune predominante del cuerpo humano, de linfocitos T colaboradores 1 tipo-Th1 a tipo-Th2. Por ello, se espera ampliamente que las infecciones parasitarias aceleren la progresión de infecciones del virus de inmunodeficiencia humana (VIH) a síndrome de inmunodeficiencia adquirida (SIDA). En la República Popular China, tanto las enfermedades parasitarias como el SIDA son epidémicos en ciertas áreas rurales, y las co-infecciones son relativamente comunes. Sin embargo, ningún estudio basado en la población había investigado aún la frecuencia de co-infecciones de parásitos y VIH, y sus efectos sobre las respuestas inmunes. Estudiamos (1) el estatus inmune de una población infectada con VIH, y (2) el efecto de co-infección de VIH y parásitos intestinales sobre parámetros seleccionados del sistema inmune humano.

Métodos: Un total de 309 individuos infectados con VIH fueron reclutados y comparados con un grupo de control de igual edad y género de 315 individuos locales VIH-negativo. Cuestionarios fueron administrados a todos los participantes para obtener información sobre características socio-demográficas, hábitos sanitarios, ingreso familiar, y manifestaciones clínicas recientes. Dos muestras consecutivas de deposiciones y muestras de 10ml de sangre venosa también fueron recolectadas de cada individuo para el diagnóstico de infecciones parasitarias y mediciones cuantitativas de citocinas y linfocitos T CD4+, respectivamente.

Resultados: Durante el período del estudio, 79 individuos infectados con VIH no estuvieron bajo terapia antirretroviral de gran actividad (TARGA) y fueron por ello incluidos en nuestro análisis; la prevalencia de infecciones helmínticas intestinales fue de 6,3% y la de protozoos fue 22,8%. Las infecciones de protozoos más comunes fueron *Blastocystis hominis (B. hominis)* (13,9%) y *Cryptosporidium* spp. (10,1%). La prevalencia de *Cryptosporidium* spp. en individuos infectados con VIH fue significativamente mayor que la de individuos VIH negativos ($P<0.05$). Comparados con la población no-co-infectada, no se observó diferencia

significativa para ninguno de los indicadores inmunológicos medidos ($P > 0.05$). Sin embargo, las siguientes tendencias fueron observadas: los niveles de IFN-γ fueron más bajos, pero el nivel IL-4 fue más alto, en la población co-infectada con VIH y helmintos. En la población co-infectada con VIH y *B. hominis*, el nivel IL-2 fue más alto. La población co-infectada con VIH y *Cryptosporidium* spp. tuvo conteos de linfocitos-T CD4+ considerablemente más bajos.

Conclusión: Según el perfil inmunológico, la co-infección con helmintos es desventajosa para individuos infectados con VIH. Estuvo asociada a un cambio en el equilibrio Th1/Th2 en la misma dirección que la causada por el virus mismo, lo que puede indicar una aceleración del progreso de una infección de VIH a SIDA. La co-infección con *Cryptosporidium* spp. no se estuvo asociada a un cambio significativo en factores inmunes pero la co-infección con *Cryptosporidium* spp. estuvo asociada a un nivel reducido de linfocitos T CD4+, confirmando la naturaleza oportunista de dichas infecciones. La co-infección con *B. hominis*, por otro lado, estuvo asociada a un cambio antagonístico en el perfil inmunológico comparado a una infección de VIH.

Translated from English version into Spanish by Denise Tarud, through

TRANSLATORS
WITHOUT BORDERS
